# NMDA receptor-dependent presynaptic inhibition at the calyx of Held synapse of rat pups

**DOI:** 10.1098/rsob.170032

**Published:** 2017-07-26

**Authors:** Tomoko Oshima-Takago, Hideki Takago

**Affiliations:** 1 Department of Rehabilitation for Sensory Functions, Research Institute, National Rehabilitation Center for Persons with Disabilities, Saitama 359-8555, Japan; 2 Department of Neurophysiology, University of Tokyo Graduate School of Medicine, Tokyo 113-0033, Japan; 3 Department of Otolaryngology, Tokyo Medical and Dental University Graduate School, Tokyo 113-8510, Japan

**Keywords:** presynaptic NMDA receptor, calcium channel, excitatory postsynaptic current, synapse, glutamate

## Abstract

*N*-Methyl-d-aspartate receptors (NMDARs) play diverse roles in synaptic transmission, synaptic plasticity, neuronal development and neurological diseases. In addition to their postsynaptic expression, NMDARs are also expressed in presynaptic terminals at some central synapses, and their activation modulates transmitter release. However, the regulatory mechanisms of NMDAR-dependent synaptic transmission remain largely unknown. In the present study, we demonstrated that activation of NMDARs in a nerve terminal at a central glutamatergic synapse inhibits presynaptic Ca^2+^ currents (I_Ca_) in a GluN2C/2D subunit-dependent manner, thereby decreasing nerve-evoked excitatory postsynaptic currents. Neither presynaptically loaded fast Ca^2+^ chelator BAPTA nor non-hydrolysable GTP analogue GTPγS affected NMDAR-mediated I_Ca_ inhibition. In the presence of a glutamate uptake blocker, the decline in I_Ca_ amplitude evoked by repetitive depolarizing pulses at 20 Hz was attenuated by an NMDAR competitive antagonist, suggesting that endogenous glutamate has a potential to activate presynaptic NMDARs. Moreover, NMDA-induced inward currents at a negative holding potential (−80 mV) were abolished by intra-terminal loading of the NMDAR open channel blocker MK-801, indicating functional expression of presynaptic NMDARs. We conclude that presynaptic NMDARs can attenuate glutamate release by inhibiting voltage-gated Ca^2+^ channels at a relay synapse in the immature rat auditory brainstem.

## 1. Introduction

The *N*-methyl-d-aspartate receptor (NMDAR), a member of the ionotropic glutamate receptor family, consists of glycine-binding GluN1 (formerly NR1) subunits together with glutamate-binding GluN2 (GluN2A–D, formerly NR2A–D) subunits and/or glycine-binding GluN3 (GluN3A,B, formerly NR3A,B) subunits, which form a heteromeric receptor complex [1,2]. Postsynaptic NMDARs show variable functions in synaptic transmission, synaptic plasticity, neuronal development and neuronal diseases (for review see [3–7]). Interestingly, over the past two decades, accumulating evidence has indicated that NMDARs are also presynaptically expressed in the cerebral cortex [8–12], hippocampus [13,14], amygdala [15], cerebellum [16–18] and spinal cord [19,20]. Activation of presynaptic NMDARs enhances spontaneous release at glutamatergic synapses in the cerebral cortex [10,21–23], hippocampus [14,24] and amygdala [25] as well as at GABAergic synapses in the cerebellum [18,26–30] and hippocampus [31]. Further, presynaptic NMDAR activation facilitates action potential-evoked glutamate release at cortical [21,22] and hippocampal [32] synapses, and induces long-term potentiation at glutamatergic synapses in the amygdala [33] and subiculum [34,35]. In contrast, previous research has also indicated that presynaptic NMDARs attenuate action potential-evoked transmitter release at both excitatory [36] and inhibitory [26,28] synapses, and mediate long-term depression of excitatory [10,17,22,37–39] and inhibitory [40] synaptic transmission. However, the mechanisms underlying presynaptic NMDAR-mediated regulation of synaptic transmission remains to be clarified.

Despite these findings, recent studies have challenged the existence of axonal/presynaptic NMDA receptors. In a previous study, focal iontophoretic application of the NMDAR agonist l-aspartate onto the axons of cerebellar stellate cells failed to elicit Ca^2+^ transients in axonal varicosities. However, l-aspartate application onto the dendrites of these cells elicited Ca^2+^ transients in axonal varicosities via opening of voltage-gated Ca^2+^ channels (VGCCs) triggered by passive propagation of depolarization from somatodendritic sites down along axons [41]. Subsequent studies by the same research group revealed no evidence of functional NMDAR expression in the axons of L5 pyramidal cells in the visual cortex [42], or basket cells in the cerebellum [43]. Given such controversial findings, presynaptic recordings should be used to explore whether other types of cells in the CNS exhibit axonal/presynaptic NMDAR expression.

At the calyx of Held synapse in the rat auditory brainstem, whose presynaptic structure is large enough to enable direct whole-cell patch-clamp recordings, application of exogenous l-glutamate inhibits nerve-evoked release of the endogenous neurotransmitter glutamate [44]. This presynaptic inhibitory action is mediated by metabotropic glutamate receptors (mGluRs) [45] and also by α-amino-3-hydroxy-5-methyl-4-isoxazolepropionic acid receptors (AMPARs) [44] expressed in the presynaptic terminal. Both types of glutamate receptors inhibit VGCCs via the activation of heterotrimeric G proteins. However, mGluR and AMPA/kainate receptor antagonists only partially impair the inhibitory effect of l-glutamate on presynaptic Ca^2+^ currents (I_Ca_), suggesting the involvement of additional mechanisms. In the present study, we show that activation of presynaptic NMDARs induces inward currents at a negative holding potential, inhibits I_Ca_ and decreases action potential-dependent excitatory postsynaptic currents (EPSCs) at the calyx of Held synapse in the immature rat brainstem.

## 2. Material and methods

### 2.1. Animals, preparations and solutions

Wistar rats (7–9 days old) of either sex were used. After decapitation under deep isoflurane or halothane inhalation anaesthesia, the brain was quickly removed. Transverse brainstem slices (200–250 µm in thickness) containing the medial nucleus of trapezoid body (MNTB) were cut ice-cold using a tissue slicer (PRO-7, Dosaka, Kyoto, Japan or VT-1200S, Leica, Mannheim, Germany) as described previously [46]. Slices were incubated at 37°C for 30 min and subsequently maintained at room temperature (21–25°C) in artificial cerebrospinal fluid (aCSF) containing (in mM): 125 NaCl, 2.5 KCl, 26 NaHCO_3_, 1.25 NaH_2_PO_4_, 2 CaCl_2_, 1 MgCl_2_, 10 d-glucose, 3 myo-inositol, 2 sodium pyruvate and 0.5 sodium ascorbate (pH 7.4 when bubbled with 95% O_2_ and 5% CO_2_). Calyces and MNTB neurons were visualized with a 60× water immersion objective lens (Olympus, Tokyo, Japan) attached to an upright microscope (BX51WI, Olympus, Tokyo, Japan or Axioskop, Zeiss, Oberkochen, Germany). For recording presynaptic Ca^2+^ currents (I_Ca_), the aCSF additionally contained tetrodotoxin (TTX, 1 µM, Wako, Osaka, Japan) plus tetraethylammonium chloride (TEACl, 10 mM; equimolar replacement for NaCl), and the presynaptic pipette solution contained (in mM): 110 CsCl, 10 TEACl, 40 HEPES, 0.5 EGTA, 1 MgCl_2_, 12 Na_2_ phosphocreatine, 2 ATP-Mg and 0.5 GTP-Na (pH 7.3 with CsOH, 295–305 mOsm kg^−1^). For recording presynaptic Ba^2+^ currents (I_Ba_), CaCl_2_ (2 mM) in the aCSF was replaced with equimolar BaCl_2_. For recording presynaptic membrane currents, the aCSF additionally contained TTX (1 µM), and the presynaptic pipette solution contained (in mM): 97.5 potassium gluconate, 32.5 KCl, 10 HEPES, 5 EGTA, 1 MgCl_2_, 12 Na_2_ phosphocreatine, 2 ATP-Mg and 0.5 GTP-Na (pH 7.3 with KOH, 295–305 mOsm kg^−1^). For recording EPSCs) the aCSF routinely contained bicuculline methiodide (10 µM, Sigma, St. Louis, MO, USA) and strychnine hydrochloride (0.5 µM, Sigma) to block GABAergic and glycinergic inhibitory synaptic currents, respectively. The postsynaptic pipette solution contained (in mM): 110 CsF, 30 CsCl, 10 HEPES, 5 EGTA, and 1 MgCl_2_ (pH adjusted to 7.3 with CsOH, 295–305 mOsm kg^−1^). Further, *N*-(2,6-diethylphenylcarbamoylmethyl)-triethyl-ammonium chloride (QX314, 5 mM, Alomone Labs, Jerusalem, Israel) was also included in the postsynaptic pipette solution to block action potential generation.

### 2.2. Chemical compounds

In addition to chemicals already mentioned above, we used the following NMDAR agonists: NMDA from Sigma and (3-chlorophenyl) [3,4-dihydro-6,7-dimethoxy-1-[(4-methoxyphenoxy)methyl]-2(1H)-isoquinolinyl]methanone (CIQ) from Tocris Bioscience (Bristol, UK). We also used the following NMDAR antagonists: 7-chlorokynurenate (7-ClK), d-(–)-2-amino-5-phosphonopentanic acid (d-AP5), 4-(5-(4-bromophenyl)-3-(6-methyl-2-oxo-4-phenyl-1,2-dihydroquinolin-3-yl)-4,5-dihydro-1H-pyrazol-1-yl)-4-oxobutanoic acid (DQP 1105), and (R,S)-α-(4-hydroxyphenyl)-β-methyl-4-(phenylmethyl)-1-piperidinepropanol (Ro 25-6981) from Tocris Bioscience as well as (+)-5-methyl-10,11-dihydro-5H-dibenzo[a,d]cyclohepten-5,10-imine maleate (MK-801) and ZnCl_2_ from Sigma. Other than NMDAR agonists/antagonists, we used dl-threo-β-benzyloxyaspartic acid (dl-TBOA) from Tocris Bioscience, BAPTA from Dojindo (Kumamoto, Japan), and tricine from Nacalai Tesque (Kyoto, Japan). We obtained all of remaining chemicals used in this study from Sigma.

### 2.3. Electrophysiological recordings

Whole-cell patch-clamp recordings were made from presynaptic calyceal nerve terminals or postsynaptic MNTB principal neurons. For recording I_Ca_ and I_Ba_, calyces were voltage-clamped at a holding potential of –80 mV, and depolarizing voltage steps (duration: 3 ms) were applied every 20 s. The I_Ca_ amplitude was measured 2–3 ms after the onset of depolarizing pulses. For recording evoked EPSCs, MNTB neurons were voltage-clamped at a holding potential of −70 mV, and presynaptic axons were stimulated every 20 s by using a tungsten bipolar electrode positioned halfway between the midline and the MNTB. The EPSC amplitude was measured at their peaks. For presynaptic recordings, the electrode resistance was 4–8 MΩ, and the access resistance was 5–18 MΩ with its compensation by 80%. Leak currents in presynaptic recordings were subtracted by the scaled pulse (P/8) protocol. For postsynaptic recordings, the electrode resistance was 2.5–4 MΩ, and access resistance was 5–15 MΩ with its compensation by 70%. Voltage-clamp recordings were made using a patch-clamp amplifier (Axopatch-200B, Axon Instruments, Foster City, CA, USA). Current-clamp recordings of presynaptic action potentials were made using another patch-clamp amplifier (MultiClamp 700A, Axon Instruments) equipped with a high input impedance (10^11^ Ω) voltage follower. Recorded signals were low-pass-filtered at 5 kHz and digitized at 20–50 kHz by an analogue–digital converter (Digidata 1322A, Axon Instruments) with pCLAMP 9 software (Axon Instruments). Liquid–junction potentials between the pipette solutions and the aCSF were not corrected for. Drugs were bath-applied by switching superfusates using a peristaltic pump or a gravity-fed perfusion system (perfusion rate, 2.0–6.0 ml min^−1^). Experiments were carried out at room temperature (21–25°C).

### 2.4. Statistical analysis

Data are presented as mean ± s.e.m.. For comparison of paired data from one group, we first used Shapiro–Wilk normality test, then employed Student's paired *t*-test. For comparison of data from the control groups and groups with various kinds of manipulations such as extracellular/intracellular application of NMDAR agonists/antagonists, we first used Shapiro–Wilk normality test, then employed Student's unpaired *t*-test. Since all the data in this study passed the normality test, nonparametric statistical analysis was not necessary. Unless otherwise described, Student's unpaired *t*-test was employed. Statistical significance was considered when *p*-value was less than 0.05 (SigmaPlot 12.0, Systat Software Inc., San Jose, CA, USA), and significance level is denoted using asterisks (*p < 0.05, **p < 0.01 and ***p < 0.001).

## 3. Results

### 3.1. Inhibitory effect of NMDA on presynaptic Ca^2+^ currents (I_Ca_)

**Figure 1.**
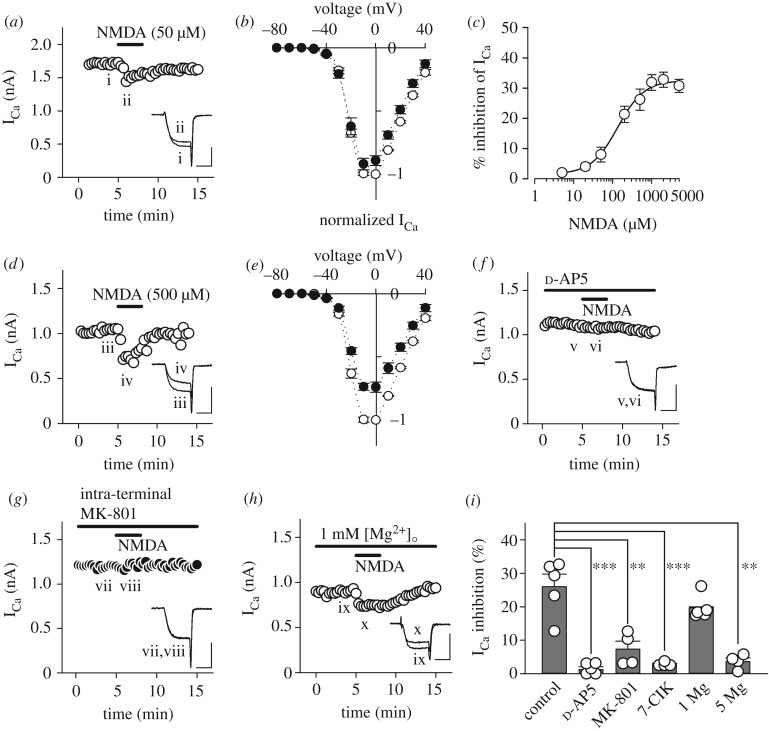
Inhibition of presynaptic Ca^2+^ currents by NMDA. (*a*) NMDA (50 µM) attenuated presynaptic Ca^2+^ currents (I_Ca_) evoked by a depolarizing pulse (from a holding potential of −80 to 0 mV (duration: 3 ms) in 0 mM Mg^2+^ aCSF. Sample records show I_Ca_ before (i) and during (ii) NMDA application. Three consecutive I_Ca_ were averaged and superimposed for each. (*b*) The current–voltage relationships of I_Ca_ before (open circles) and during (filled circles) 50 µM NMDA application. Mean amplitude of I_Ca_ from 5 calyces at each membrane potential was normalized to that at 0 mV before NMDA application. (*c*) The concentration-dependence of NMDA-induced I_Ca_ inhibition. Individual data points and bars indicate mean ± s.e.m. derived from 4–5 calyces. IC_50_ value was 135 µM. (*d*) I_Ca_ inhibition by NMDA (500 µM) in 0 mM Mg^2+^ aCSF as a control for comparison with that in the presence of NMDAR antagonists. Sample records of I_Ca_ before (iii) and during (iv) NMDA application. (*e*) The current–voltage relationships of I_Ca_ before (open circles) and during (filled circles) 500 µM NMDA application. (*f*) Bath-application of d-AP5 (500 µM) blocked NMDA-induced I_Ca_ inhibition (v,vi). (*g*) MK-801 (500 µM) loaded into the presynaptic terminal nearly abolished NMDA-induced I_Ca_ inhibition (vii,viii). (*h*) Physiological concentration of extracellular Mg^2+^ (1 mM) only weakly attenuated NMDA-induced I_Ca_ inhibition (ix,x). (*i*) Summary of percentage inhibition of I_Ca_ by NMDA (500 µM) in the absence (control, *n* = 5) or presence of the NMDAR blockers such as d-AP5 (500 µM, *n* = 5), intra-terminal MK-801 (iMK-801, 500 µM loaded into presynaptic terminals, *n* = 4) and 7-ClK (100 µM, *n* = 4) as well as those in the presence of Mg^2+^ (1 mM, *n* = 5; 5 mM, *n* = 4). Both individual data (open circles) and mean ± s.e.m. (dark grey bars) are shown. Asterisks indicate significant statistical differences (**p < 0.01, ***p < 0.001). Scale bars in the superimposed sample traces indicate 2 ms for horizontal and 1 nA for vertical axes, respectively.

Previous studies have revealed that activation of mGluRs [45] or AMPARs [44] in the calyx of Held presynaptic terminal inhibits I_Ca_. First, we investigated whether NMDA also inhibits I_Ca_. As illustrated in figure 1*a*, bath-application of NMDA (50 µM) in Mg^2+^-free aCSF inhibited I_Ca_ by 8.3 ± 2.7% at 0 mV (*n* = 5) without a clear shift in the current–voltage relationship (figure 1*b*). The inhibitory effect of NMDA on I_Ca_ was concentration-dependent with an IC_50_ of 135 µM (figure 1*c*). Based on this result, we used a relatively high concentration of NMDA (500 µM) for controls in order to securely analyse the property of NMDA-induced I_Ca_ inhibition. As illustrated in figure 1*d*, bath-application of NMDA at this concentration in Mg^2+^-free aCSF more evidently inhibited I_Ca_ by 26.0 ± 3.7% at 0 mV (*n* = 5), again without a clear shift in the current–voltage relationship (figure 1*e*). The NMDAR competitive antagonist d-AP5 (500 µM) abolished this NMDA effect (to 1.2 ± 0.9%, *n* = 5, ***p < 0.001; figure 1*f*,*i*). Although glycine (10 µM) had no effect on NMDA-induced I_Ca_ inhibition (29.9 ± 2.8%, *n* = 5, *p* = 0.222), the NMDAR glycine-site blocker 7-chlorokynurenic acid (7-ClK, 100 µM) blocked it (to 2.8 ± 0.3%, *n* = 4, ***p < 0.001; figure 1*i*), suggesting that glycine sites of NMDARs may be saturated by endogenous ligand(s) in slices. Surprisingly, a physiological concentration of extracellular Mg^2+^ (1 mM) did not significantly weaken NMDA-induced I_Ca_ inhibition (19.7 ± 1.6%, *n* = 5, *p* = 0.158; figure 1*h*,*i*), although a higher concentration of extracellular Mg^2+^ (5 mM) successfully abolished the inhibition (to 3.6 ± 1.1%, *n* = 4, **p < 0.01; figure 1*i*). These results indicate that NMDA-induced I_Ca_ inhibition was indeed mediated by NMDARs. To exclude the possibility that NMDARs expressed in surrounding neurons and/or glia might mediate this NMDA effect, we loaded the NMDAR open channel blocker MK-801 (500 µM) directly into the calyceal nerve terminal through a whole-cell patch pipette. We set the concentration of intra-terminal MK-801 to a range of sub-millimolar based on the procedure used in a previous study, in which MK-801 (1 mM) was applied into presynaptic neurons through the patch pipettes [38]. Under this condition, intra-terminal MK-801 nearly abolished NMDA-induced I_Ca_ inhibition (to 7.3 ± 2.4%, *n* = 4, **p < 0.01; figure 1*g*,*i*), suggesting that functional NMDARs are expressed in calyceal nerve terminals, and that their activation inhibits presynaptic VGCCs.

### 3.2. Subunit dependence of NMDAR-mediated I_Ca_ inhibition

**Figure 2.**
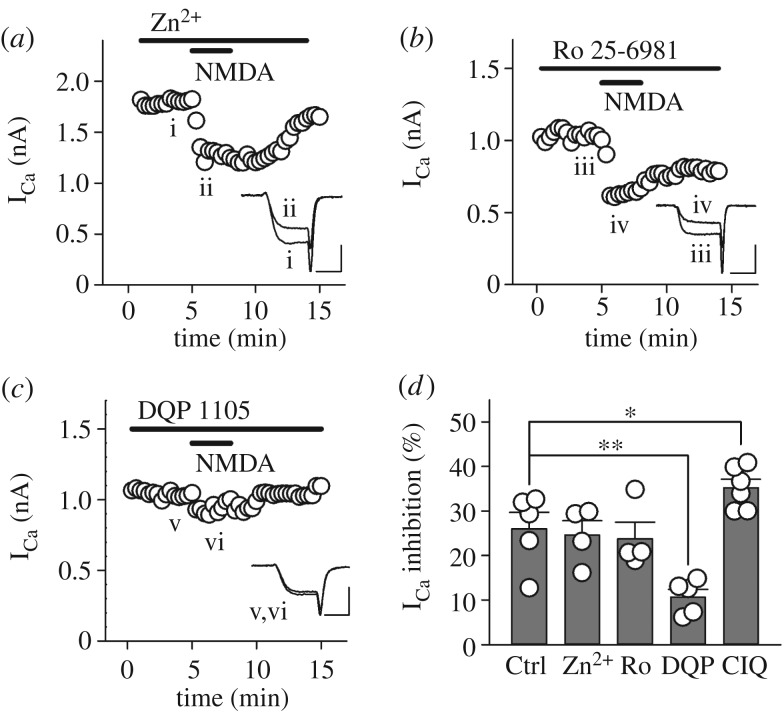
NMDAR-mediated I_Ca_ inhibition is GluN2C/2D-dependent. In each experiment, the bath superfusate additionally contained one of the specific NMDAR subunit antagonists: (*a*) zinc (300 nM as free Zn^2+^) for GluN2A, (*b*) Ro 25-6981 (1 µM) for GluN2B, and (*c*) DQP 1105 (10 µM) for GluN2C/2D, respectively. (*d*) Summary of NMDAR subunit dependence of I_Ca_ inhibition. Note that a GluN2C/2D potentiator, CIQ (10 µM), augmented NMDAR-mediated I_Ca_ inhibition. Both individual data (open circles) and mean ± s.e.m. (dark grey bars) are shown. Asterisks indicate significant statistical differences (*p < 0.05, **p < 0.01). Scale bars in the superimposed sample traces indicate 2 ms for horizontal and 1 nA for vertical axes, respectively.

We next investigated which NMDAR subunits contribute to NMDA-induced inhibition of presynaptic VGCCs. Since sub-micromolar concentrations of Zn^2+^ selectively blocks the GluN2A subunit [47], we examined the effect of Zn^2+^ on NMDA-induced I_Ca_ inhibition. In the presence of 300 nM of free Zn^2+^ in the superfusate, which was achieved by a combination of ZnCl_2_ (27 µM) and zinc buffer tricine (10 mM), NMDA (500 µM) still inhibited I_Ca_ to a similar extent as the control (24.6 ± 3.2%, *n* = 4, *p* = 0.792; figure 2*a*,*d*). For the GluN2B subunit, we used the GluN2B selective antagonist Ro 25-6981 (1 µM) and found no significant difference compared with the control (23.8 ± 3.7%, *n* = 4, *p* = 0.690; figure 2*b*,*d*). In contrast, the GluN2C/2D selective antagonist DQP 1105 (10 µM) significantly weakened the NMDA-induced I_Ca_ inhibition (10.7 ± 1.7%, *n* = 5, **p < 0.01; figure 2*c*,*d*), whereas the GluN2C/2D selective potentiator CIQ (10 µM) significantly strengthened this inhibition (35.2 ± 1.9%, *n* = 6, *p < 0.05; figure 2*d*). We were unable to pharmacologically evaluate the involvement of the GluN3 subunit due to the lack of subunit-specific agonists/antagonists. Taken these results together, GluN2C/2D subunits, but not GluN2A or GluN2B subunits, contribute to NMDAR-mediated inhibition of presynaptic VGCCs at the immature calyx of Held synapse.

### 3.3. G proteins and Ca^2+^ are dispensable for NMDAR-mediated I_Ca_ inhibition

**Figure 3.**
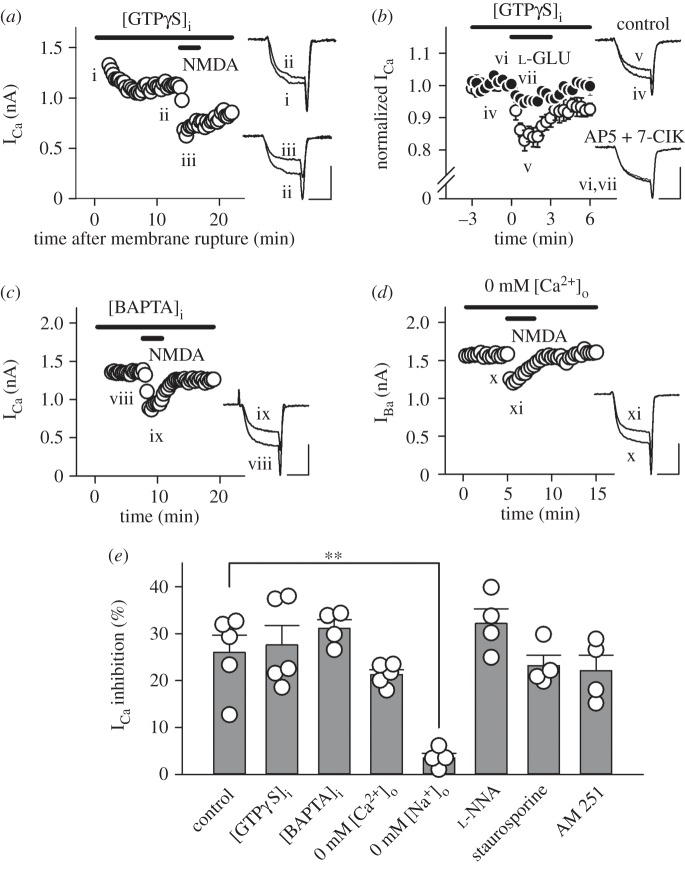
NMDAR-mediated I_Ca_ inhibition requires neither G proteins nor Ca^2+^. (*a*) Intra-terminal loading of GTPγS (0.2 mM) attenuated I_Ca_ (i,ii), but had no effect on NMDA-induced I_Ca_ inhibition (iii). (*b*) In the presence of GTPγS (0.2 mM), l-glutamate (500 µM) inhibited I_Ca_ (open circles, iv,v, *n* = 4). A cocktail of NMDAR blockers (500 μM d-AP5 plus 100 μM 7-ClK) weakened the l-glutamate-induced I_Ca_ inhibition (filled circles, vi,vii, *n* = 4). (*c*) NMDA (500 µM) attenuated I_Ca_ (viii, ix) in the presence of BAPTA (10 mM) in the presynaptic terminal. (*d*) NMDA (500 µM) attenuated I_Ba_ (x,xi) through presynaptic Ca^2+^ channels. I_Ba_ was evoked by a depolarizing pulse to 0 mV (duration: 3 ms). Scale bars in the superimposed sample traces indicate 2 ms for horizontal and 1 nA for vertical axes, respectively. (*e*) Summary of percentage inhibition of I_Ca_ by NMDA (500 µM) in the absence (control, *n* = 5) or presence of the various agents to explore a candidate intracellular mechanism(s) which underlies NMDAR-mediated I_Ca_ inhibition. In addition to intra-terminal GTPγS ([GTPγS]_i_, *n* = 5) and BAPTA ([BAPTA]_i_, *n* = 4) as well as replacement of extracellular Ca^2+^ with Ba^2+^ (0 mM [Ca^2+^]_o_, *n* = 5), omission of extracellular Na (0 mM [Na^+^]_o_, *n* = 4), nitric oxide synthesis inhibitor l-NNA (1 mM, *n* = 4), protein kinase C inhibitor staurosporine (2 µM, *n* = 4), and cannabinoid receptor type 1 inhibitor AM 251 (5 µM, *n* = 4) were tested. Asterisks indicate a significant statistical difference (**p < 0.01).

At the calyx of Held, a variety of presynaptic receptors are coupled to the heterotrimeric G proteins, and direct interaction of Gβγ subunits with presynaptic VGCCs inhibits I_Ca_ as shown for mGluRs [45], GABA_B_Rs [48,49], noradrenaline α_2_Rs [50], adenosine A_1_Rs [51], 5-HT_1B_Rs [52] and AMPARs [44]. To investigate the possibility that this mechanism also underlies NMDA-induced I_Ca_ inhibition, we loaded the non-hydrolysable GTP analogue GTPγS (0.2 mM) into the presynaptic terminal through whole-cell patch pipettes. As GTPγS diffused into a terminal from a presynaptic pipette, I_Ca_ became smaller in amplitude and slower in rise time, consistent with previous studies [48,53]. After the I_Ca_ amplitude had reached a steady level, bath-application of NMDA (500 µM) attenuated I_Ca_ (figure 3*a*,*e*) by 27.5 ± 4.2% (*n* = 5). This magnitude of inhibition in the presence of intra-terminal GTPγS was similar to that observed in its absence (*p* = 0.784), suggesting that NMDAR-mediated I_Ca_ inhibition does not require G proteins. We then sought to confirm that the lack of occlusive effect of intra-terminal GTPγS on NMDA-induced I_Ca_ inhibition was not due to a failure of drug action. Following bath-application of the high affinity group III mGluR agonist l-AP4 (100 µM), significant differences in the magnitude of I_Ca_ inhibition were observed between the absence (22.5 ± 1.6%, *n* = 5) and presence (2.2 ± 1.3%, *n* = 5, ***p < 0.001, data not shown) of intra-terminal GTPγS (0.2 mM). Thus, intra-terminal GTPγS securely occluded mGluR-mediated I_Ca_ inhibition. After intra-terminal GTPγS (0.2 mM) had fully activated the mGluR- and AMPAR-mediated I_Ca_ inhibition pathways, we examined whether l-glutamate further inhibits I_Ca_ via the activation of presynaptic NMDARs. As shown in figure 3*b*, bath-application of l-glutamate (500 µM) inhibited I_Ca_ by 19.4 ± 0.6% (*n* = 4), and this effect was lessened to 6.1 ± 0.9% (*n* = 4, *p < 0.05) by a mixture of d-AP5 (500 µM) and 7-ClK (100 µM). These results suggest that presynaptic NMDARs mainly mediate l-glutamate-induced additional I_Ca_ inhibition after full activation of mGluRs and AMPARs [44].

We then examined whether intra-terminal Ca^2+^, which is elevated by presynaptic NMDAR activation, mediates NMDA-induced I_Ca_ inhibition. The fast Ca^2+^ chelator BAPTA (10 mM) loaded into the calyceal terminal had no effect on NMDA-induced I_Ca_ inhibition (31.1 ± 1.8%, *n* = 4, *p* = 0.290; figure 3*c*,*e*). Moreover, replacement of the VGCC charge carrier Ca^2+^ with Ba^2+^ (2 mM) had no significant effect on the NMDA-induced inhibition of presynaptic Ba^2+^ currents (21.2 ± 1.0%, *n* = 5, *p* = 0.254; figure 3*d*,*e*). These results suggest that intra-terminal Ca^2+^ does not contribute to NMDA-induced I_Ca_ inhibition.

We also examined a possible effect of Na^+^ on the I_Ca_ inhibition. When extracellular Na^+^ was replaced with equimolar TEA^+^, bath-application of NMDA (500 µM) no longer inhibited I_Ca_ (3.5 ± 1.0%, *n* = 4; **p < 0.01; figure 3*e*), suggesting that Na^+^ influx through presynaptic NMDARs may somehow mediate the I_Ca_ inhibition.

We further aimed to identify the intracellular mechanism(s) that links NMDAR activation and Ca^2+^ channel inhibition in the calyceal terminal. Since presynaptic NMDARs are relevant to nitric oxide synthesis in the cerebellum [54,55] as well as protein kinase C activation in the neocortex [56], we examined whether such chemicals as the nitric oxide synthase inhibitor l-NNA (1 mM) or the protein kinase C inhibitor staurosporine (2 µM) attenuate I_Ca_ inhibition induced by NMDA (500 µM). However, neither agent exerted a significant effect (32.1 ± 3.1%, *n* = 4, *p* = 0.259 for l-NNA; 23.1 ± 2.3%, *n* = 4, *p* = 0.560 for staurosporine, figure 3*e*). Moreover, we examined whether endocannabinoid signalling triggered via the activation of NMDARs in the postsynaptic MNTB neuron is associated with NMDA-induced I_Ca_ inhibition. Based on the protocol in a previous study [57], we performed these experiments using the cannabinoid receptor type 1 blocker AM 251 (5 µM). In the presence of AM 251 in aCSF, NMDA application still inhibited I_Ca_ by 23.6 ± 3.0% (*n* = 4, *p* = 0.473, figure 3*e*), suggesting that endocannabinoid-dependent retrograde signalling is not involved in NMDA-induced I_Ca_ inhibition.

### 3.4. NMDA-induced currents in the calyceal nerve terminal

**Figure 4.**
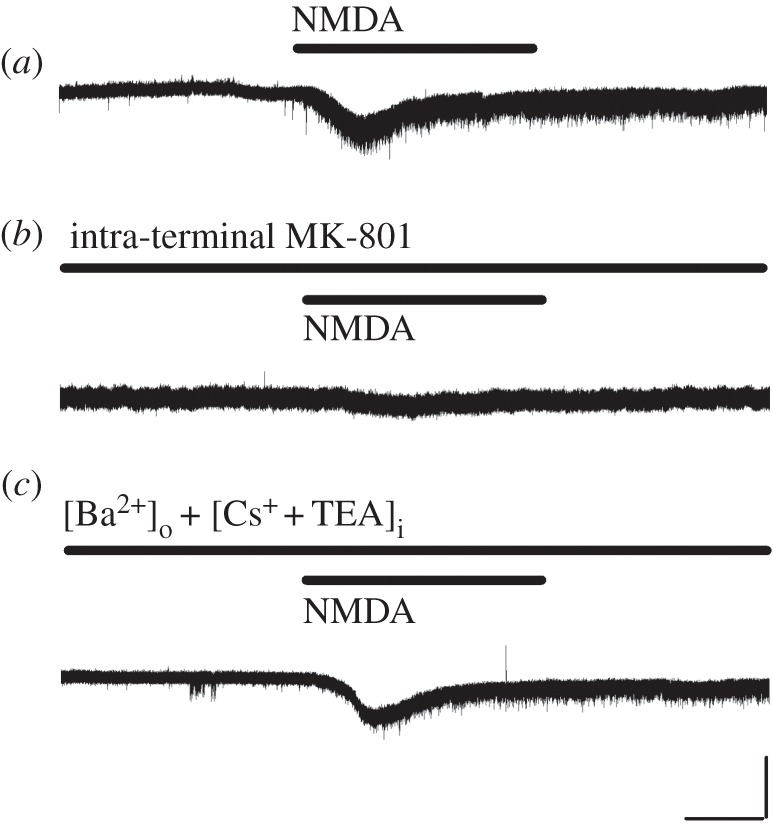
NMDA-induced currents in calyceal nerve terminals. (*a*) Bath-application of NMDA (500 µM) induced inward currents at a holding potential of −80 mV, with concomitant increase in membrane noise. (*b*) Intra-terminal MK-801 (500 µM) blocked the NMDA currents. (*c*) In the presence of intra-terminal Cs^+^ (110 mM) as well as TEA (10 mM) and Ba^2+^ (2 mM, substituted for Ca^2+^) in aCSF, NMDA (500 µM) induced significant inward currents, again with concomitant increase in membrane noise. Data are obtained in Mg^2+^-free aCSF. Scale bars in the superimposed sample traces indicate 1 min for horizontal and 20 pA for vertical axes, respectively.

We examined whether NMDARs expressed in the calyceal nerve terminal exhibit ionotropic channel properties. In Mg^2+^-free aCSF containing TTX (1 µM), at a holding potential of –80 mV, bath-application of NMDA (500 µM) induced inward currents (35.5 ± 9.9 pA, *n* = 7, figure 4*a*), which were accompanied by an increase in membrane noise (figure 4*a*). Intra-terminal MK-801 (500 µM) significantly reduced these NMDA currents to 8.9 ± 2.2 pA (*n* = 4; figure 4*b*). After maximal blockade of presynaptic K^+^ channels by using a Cs^+^-based pipette solution containing TEA (10 mM) and also replacement of extracellular Ca^2+^ with equimolar Ba^2+^ (2 mM), bath-application of NMDA (500 µM) still induced inward currents (17.3 ± 6.9 pA, *n* = 5; figure 4*c*), which were again accompanied by an increase in membrane noise (figure 4*c*). Intra-terminal MK-801 (500 µM) had no effect on the presynaptic resting membrane potential (−60.6 ± 1.2 mV for control, −61.8 ± 1.5 mV for MK-801, *n* = 5 for each, *p* = 0.471), peak amplitude (95.6 ± 2.0 mV for control, 96.8 ± 2.1 mV for MK-801, *p* = 0.655) or half-width (0.49 ± 0.04 ms for control, 0.47 ± 0.07 ms for MK-801, *p* = 0.835) of presynaptic action potentials, suggesting that its blocking effect is specific for NMDARs. These results indicate that functional NMDARs are expressed in the calyceal nerve terminals. The reduction in the inward current amplitude following blockade of K^+^ channels and small currents remaining in the presence of intra-terminal MK-801 (figure 4*b*) imply that activation of NMDARs in surrounding cells might additionally contribute to NMDA-induced inward currents via an increase in extracellular K^+^ concentration.

### 3.5. NMDAR-mediated I_Ca_ inhibition by endogenous glutamate

**Figure 5.**
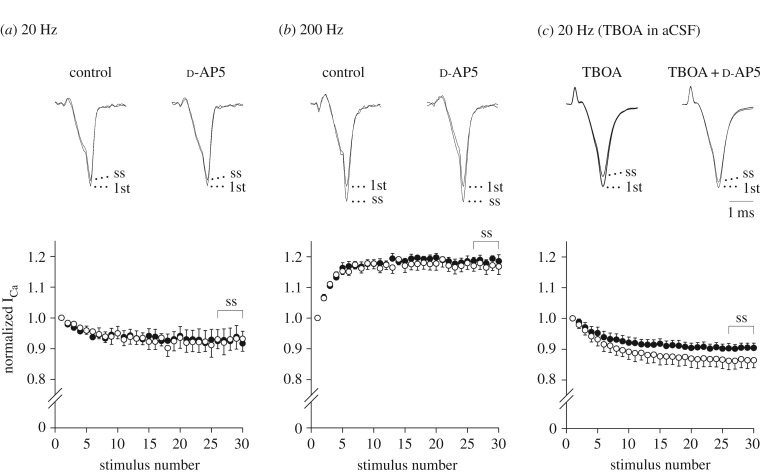
Involvement of endogenous NMDA receptor ligand(s) in activity-dependent decline of I_Ca_. I_Ca_ were elicited by a train of 30 square depolarizing pulses (to 0 mV, duration: 1 ms). Sample records show the first (1st) and 26th–30th averaged I_Ca_ (ss, superimposed), which are normalized to the first I_Ca_, in the absence (left traces) or presence (right traces) of d-AP5 (500 µM). Data points and bars represent mean ± s.e.m. of the normalized I_Ca_ amplitude in the presence (filled circles) or absence (open circles) of d-AP5 in the aCSF. (*a*) Activity-dependent decline of I_Ca_ amplitude by 20 Hz stimuli. No significant difference. (*b*) Activity-dependent facilitation of I_Ca_ amplitude by 200 Hz stimuli. No significant difference. (*c*) Activity-dependent decline of I_Ca_ amplitude by 20 Hz stimuli in the presence of the glutamate transporter inhibitor TBOA (100 µM). In the presence of d-AP5, I_Ca_ decline became significantly less (*p* < 0.05, Student's paired *t*-test). A horizontal scale bar in the superimposed sample traces indicates 1 ms.

We then investigated whether endogenous glutamate inhibits I_Ca_ via the activation of presynaptic NMDARs. When I_Ca_ were evoked by a train of 30 depolarizing pulses (to 0 mV, duration: 1 ms) at 20 Hz, the I_Ca_ amplitude displayed an activity-dependent decline, and reached a steady-state (ss) level lower than that of the first I_Ca_ (I_ss_/I_1st_: 92.9 ± 4.0%, *n* = 5). Previous research reported that elevated extracellular glutamate by repetitive depolarizing stimuli activates presynaptic mGluRs, thereby reducing glutamate release [58]. This mechanism may have partly contributed to the I_Ca_ decline that we observed in this experiment. In the presence of d-AP5 (500 µM) in aCSF, no changes in this I_Ca_ decline were observed (I_ss_/I_1st_: 92.6 ± 4.3%, *n* = 5, *p* = 0.615, Student's paired *t*-test, figure 5*a*). In contrast, when I_Ca_ were evoked by a train at a higher-frequency of 200 Hz, the I_Ca_ amplitude displayed activity-dependent facilitation as previously reported for rats [59] and for mice [60]. Under this condition, d-AP5 again failed to alter the magnitude of facilitation (I_ss_/I_1st_: 117.0 ± 2.2% for control, 118.6 ± 1.6% for d-AP5, *n* = 5, *p* = 0.536, Student's paired *t*-test, figure 5*b*).

Further, to clarify whether endogenous glutamate activates presynaptic NMDARs, I_Ca_ were evoked by a train at 20 Hz in the presence of the glutamate uptake blocker TBOA (100 µM) in aCSF. As observed in the absence of TBOA, the I_Ca_ amplitude displayed activity-dependent decline and reached a steady-state level lower than that of the first I_Ca_ (I_ss_/I_1st_: 86.4 ± 2.8%, *n* = 7). Previous studies already revealed that not only repetitive depolarizing stimuli [58] but also glutamate transporter blockade by TBOA [61] raises extracellular glutamate, thereby activating presynaptic mGluR-dependent autoinhibition of glutamate release. Both mechanisms may have partly contributed to the I_Ca_ decline that we observed with this protocol. Under this condition, bath-application of d-AP5 (500 µM) significantly weakened this magnitude of I_Ca_ decline (I_ss_/I_1st_: 90.3 ± 1.6%, *p* < 0.05, figure 5*c*). These results imply that endogenous glutamate released from the nerve terminal or surrounding cells [14,62] inhibits I_Ca_ via the activation of presynaptic NMDARs. Furthermore, these results suggest that NMDAR-mediated presynaptic inhibition may not occur under physiological conditions, in which extracellular glutamate is promptly cleared by glial uptake systems. However, this inhibition may occur under pathological conditions, in which extracellular glutamate concentration rises to a high level.

### 3.6. Reduction of evoked AMPA-EPSC amplitude by NMDA

**Figure 6.**
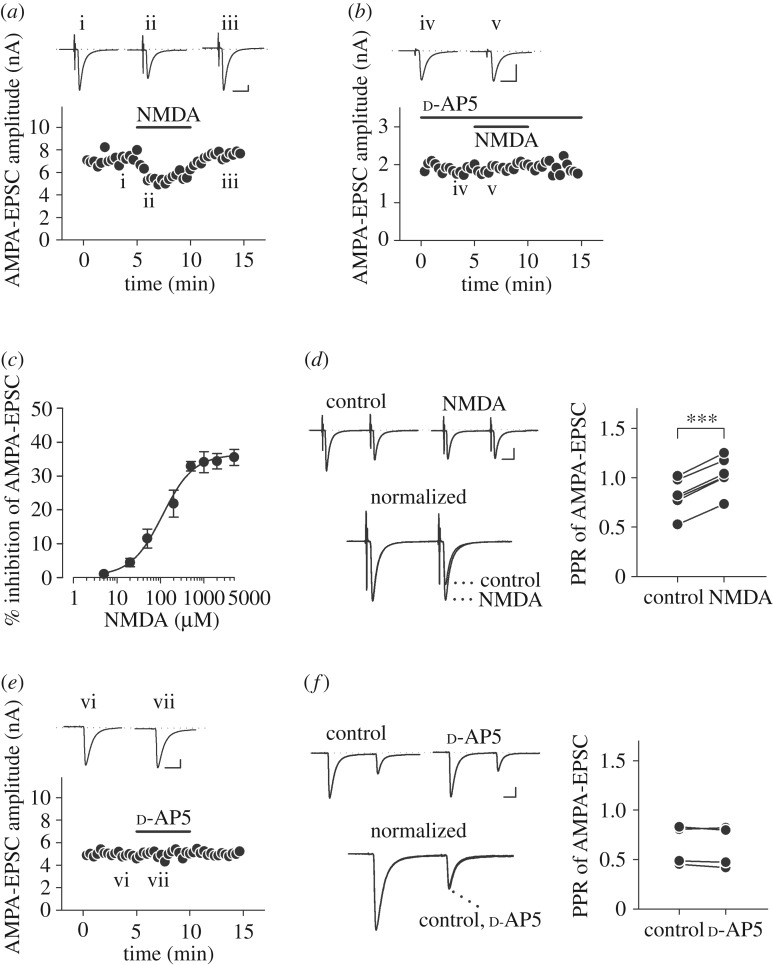
Attenuation of glutamate release by NMDA. (*a*) Inhibition of evoked AMPA-EPSCs by NMDA (500 µM) in the presence of Mg^2+^ (1 mM) in aCSF and MK-801 (5 mM) in an MNTB neuron. Note that the concentration of postsynaptically loaded MK-801 (5 mM) was 10 times higher than that of presynaptically loaded MK-801 (500 µM, figure 1) for maximal block of postsynaptic NMDARs. Sample records show AMPA-EPSCs before (i) and during (ii) bath-application of NMDA (500 µM), and after washout (iii). Three consecutive EPSCs were averaged and superimposed for each. (*b*) Bath-application of d-AP5 (500 µM) blocked the inhibitory effect of NMDA on evoked AMPA-EPSCs (iv,v). (*c*) The concentration-dependence of NMDA-induced evoked AMPA-EPSC inhibition. Individual data points and bars indicate mean ± s.e.m. derived from 4 to 7 neurons. IC_50_ value was 112 µM. (*d*) AMPA-EPSCs evoked by paired-pulse stimulation (inter-pulse interval: 20 ms) in the presence of intracellular MK-801 (5 mM). Sample records in the upper panel show AMPA-EPSCs before (control) and during NMDA application (NMDA). Those in the bottom panel show EPSCs normalized to the first amplitude, in the presence and absence of NMDA (500 µM) (superimposed). Plots on the right panel indicate PPRs before and after NMDA application in 7 neurons. NMDA significantly increased the PPR of AMPA-EPSCs (***p < 0.001, Student's paired *t*-test). (*e*) Bath-application d-AP5 (500 µM) did not alter the amplitude of AMPA-EPSCs. Sample records show AMPA-EPSCs before (vi) and during (vii) its application. (*f*) PPRs were unaffected by d-AP5 application in 4 neurons. No significant difference. Scale bars in the superimposed sample traces indicate 5 ms for horizontal and 1 nA for vertical axes, respectively.

Finally, we examined whether NMDA has an inhibitory effect on glutamatergic postsynaptic currents recorded from MNTB neurons. To abolish postsynaptic NMDA action, the NMDAR open channel blocker MK-801 (5 mM) was applied into MNTB neurons through postsynaptic patch pipettes in aCSF containing Mg^2+^ (1 mM). We set the concentration of intracellular MK-801 to 5 mM in accordance with procedures described in previous studies, which used 5 mM [24], 4 and 1 mM [39], 2 mM [14], or 1 mM [38,63,64] of intracellular MK-801. The selected concentration in this experiment was 10 times higher than that used for presynaptic recordings (500 µM) in order to ensure maximal blockade of postsynaptic NMDARs. Under this condition, bath-application of NMDA (500 µM) attenuated evoked AMPA-EPSCs (figure 6*a*) by 32.9 ± 1.4% (*n* = 7) with minimal change in holding currents in MNTB neurons (96.5 ± 38.0 pA at −70 mV). As shown in NMDA-induced I_Ca_ inhibition (figure 1*f*,*i*), the NMDAR competitive antagonist d-AP5 blocked NMDA-induced EPSC reduction (to 2.4 ± 1.4%, *n* = 4, *p* < 0.05, figure 6*b*). The inhibitory effect of NMDA on evoked AMPA-EPSCs was concentration-dependent with an IC_50_ of 112 µM (figure 6*c*). In the paired-pulse stimulation protocol with an inter-pulse interval of 20 ms, NMDA (500 µM) increased the paired-pulse ratio (PPR, the ratio of second amplitude to the first) of AMPA-EPSCs by 28.7 ± 2.6% (*n* = 7, ***p < 0.001, Student's paired *t*-test; figure 6*d*). Thus, this finding confirmed that NMDA application indeed decreases evoked AMPA-EPSCs by means of a presynaptic mechanism.

Moreover, we examined whether tonic activation of presynaptic NMDAR by endogenous glutamate alters basic synaptic transmission. Bath-application of the NMDAR competitive blocker d-AP5 (500 µM) altered neither amplitude (100.5 ± 1.8% of control, *n* = 4, *p* = 0.829, figure 6*e*) nor PPR (96.9 ± 1.9% of control, *n* = 4, *p* = 0.252, Student's paired *t*-test, figure 6*f*) of evoked AMPA-EPSCs, suggesting that presynaptic NMDARs are not tonically activated to reduce action potential-dependent release.

## 4. Discussion

In the present study, we demonstrated that activation of GluN2C/2D subunit-containing presynaptic NMDARs inhibits VGCCs, thereby attenuating action potential-driven release at a central glutamatergic synapse of young rats. Furthermore, we successfully recorded NMDA-induced currents using presynaptic voltage-clamp recordings, confirming functional expression of presynaptic NMDARs at this synapse.

Presynaptic inhibitory effects of NMDAR activation on nerve-evoked synaptic currents were reported at inhibitory synapses in the cerebellum [26,28] as well as excitatory synapses in the spinal cord primary afferents [36]. These studies reported a weak blocking effect of Mg^2+^ on NMDAR-mediated presynaptic inhibition, indicating that GluN2C/2D (preferentially GluN2D) subunits may be involved. In the present study, we also observed a weak blocking effect of Mg^2+^ on NMDAR-mediated I_Ca_ inhibition (figure 1*g*,*h*). Further, we confirmed that the GluN2C/2D selective antagonist DQP 1105 weakened (figure 2*c*,*d*) but the GluN2C/2D selective potentiator CIQ strengthened NMDAR-mediated I_Ca_ inhibition (figure 2*d*). Postsynaptic MNTB neurons before the onset of hearing employ GluN2A/2B subunit-containing NMDARs [65], whereas presynaptic calyceal terminals use GluN2C/2D subunit-containing NMDARs (figure 2*c*,*d*). Thus, NMDA induced the GluN2C/2D-dependent I_Ca_ inhibition, which may decrease the action potential-driven glutamate release, resulting in the reduction of evoked EPSCs (figure 6*a*). Notably, presynaptic NMDAR activation still inhibited VGCCs (figure 1*h*,*i*) and glutamate release (figure 6*a*) in the presence of a physiological concentration of Mg^2+^ (1 mM) in aCSF, where postsynaptic NMDARs are blocked at resting membrane potential. It is also noteworthy that postsynaptic MNTB neurons predominantly employ GluN2A/2C subunit-containing NMDARs after the onset of hearing [66].

At some other synapses, activation of presynaptic NMDARs enhances nerve-evoked transmitter release [23,67]. Whereas tonic elevation of intra-terminal Ca^2+^ facilitates transmitter release, it potentially inhibits nerve-evoked transmitter release via adaptation of Ca^2+^ sensor for exocytosis [68]. At the calyx of Held, however, either intra-terminal BAPTA (10 mM) or replacement of the charge carrier Ca^2+^ with Ba^2+^ had no effect on NMDA-induced I_Ca_ inhibition. This excludes the involvement of Ca^2+^-dependent intracellular mechanism(s). Sustained depolarization of the nerve terminal upon NMDA application may inhibit evoked transmitter release by reducing the amplitude of presynaptic action potential. However, this was not the case for NMDAR-mediated presynaptic inhibition in the present study. Expected presynaptic depolarization from the inward currents produced by NMDA (less than 50 pA) is less than 10 mV [44]. Such a mild depolarization facilitates rather than inhibits transmitter release [69,70] by causing tonic Ca^2+^ entry into the terminal [69]. However, upon activation of presynaptic NMDARs, this facilitatory effect was actually masked by stronger inhibitory effect of presynaptic NMDAR-dependent I_Ca_ inhibition. This effect may have been associated with the smaller reduction in evoked EPSC amplitude (32.9% in 1 mM Mg^2+^ in aCSF, figure 6), compared to the reduction in I_Ca_ amplitude (18.1% in 1 mM Mg^2+^ in aCSF, figure 1*h*,*i*) (cf. the EPSC amplitude is proportional to the fourth power of I_Ca_ [71]). This discrepancy may also be explained by spillover of a millimolar range of MK-801 from the patch pipette during its approach onto the postsynaptic MNTB neuron, which may have partially attenuated presynaptic NMDARs upon the EPSC recording.

Blockade of NMDA-induced I_Ca_ inhibition by loading of intra-terminal MK-801 (figure 1*g*) and by omission of extracellular Na^+^ (figure 3*e*) implies that Na^+^ influx through presynaptic NMDARs may be involved. Interestingly, Na^+^ suppressed the enhancement of spontaneous transmitter release by presynaptic NMDAR activation in the mouse primary visual cortex [56]. The Na^+^-mediated regulation mechanism should be elucidated in future studies.

The VGCC in the calyceal terminal is a common target for presynaptic G protein-coupled receptors (GPCRs), including mGluRs [45], GABA_B_Rs [48,72], noradrenaline α_2_Rs [50], adenosine A_1_Rs [51] and 5-HT_1B_Rs [52] as well as presynaptic AMPARs [44]. These receptors activate heterotrimeric G proteins, and direct interaction of Gβγ subunits with presynaptic Ca^2+^ channels inhibits I_Ca_ [49]. These presynaptic inhibitory effects of GPCR ligands on VGCCs occlude with each other [51] and are blocked by the non-hydrolysable GTP analogue GTPγS loaded into the calyceal terminal [49], implying that they share the same pathway. However, the present study showed intra-terminal GTPγS had no effect on NMDA-induced I_Ca_ inhibition. This suggests that the mechanism which links NMDARs to VGCCs is distinct from the common GTP-G protein pathway.

Then, we aimed to identify the intracellular mechanism(s) to connect NMDAR activation to VGCC inhibition in the calyceal nerve terminal using the nitric oxide synthase inhibitor l-NNA, the protein kinase C inhibitor staurosporine and the CBR1 inhibitor AM 251. However, none of these inhibitors weakened NMDA-induced I_Ca_ inhibition. Thus, future studies to determine the candidate intracellular mechanism(s) are needed.

Some pieces of evidence in the present study demonstrate functional expression of NMDARs in calyceal nerve terminals rather than in surrounding cells. First, NMDA-induced inward currents were recorded in the calyceal terminal following blockade of potassium conductance (figure 4*c*). Second, loading of MK-801 into the nerve terminal blocked NMDA-induced inhibition of I_Ca_ (figure 1*g*) and NMDA-induced inward currents (figure 4*b*), whereas NMDA-induced presynaptic inhibition was observed in the presence of MK-801 in postsynaptic MNTB cells (figure 6*a*). Third, after replacement of extracellular Ca^2+^ with Ba^2+^, NMDA inhibited presynaptic Ba^2+^ currents (figure 3*d*), despite the fact that synaptic transmission is nearly terminated after replacement of Ca^2+^ with Ba^2+^ [44]. Unfortunately, the extremely low amplitude of NMDA-induced presynaptic membrane currents (17.3 pA at −80 mV, figure 5*c*) prevented us from further dissecting the properties of NMDARs expressed in calyceal terminals.

The presynaptic inhibitory effect of NMDA had an IC_50_ of 135 µM for I_Ca_ (figure 1*c*) and 112 µM for evoked EPSCs (figure 6*c*), respectively. The recombinant NMDAR currents in *Xenopus* oocytes have an EC_50_ of 30–60 µM for NMDA, depending on GluN2 subunits co-expressed with the GluN1 subunit [73]. Similarly, the EC_50_ of native NMDAR currents in CA1 neurons in hippocampal slices is 38 µM [74]. The relatively low affinity of presynaptic NMDARs may reflect the involvement of GluN1 splice variants with low ligand affinity [75]. Ambient glutamate concentration is 55 nM at the calyx of Held synapse of immature rats [76]. In hippocampal slices, the glutamate concentration is 30 nM in the absence of the glutamate transporter inhibitor TBOA, but rises to 200 nM in its presence [74,77]. In the synaptic cleft, the glutamate concentration is estimated to rise above 300 µM [78] or up to 1 mM [79] during excitatory transmission. In experimental anoxia, the glutamate concentration in the cerebral cortex can rise to 400 µM *in vivo* [80]. Our finding that d-AP5 affected I_Ca_ evoked by repetitive stimulation in the presence of TBOA, but not in its absence (figure 5), suggests that presynaptic NMDARs do not operate under physiological conditions. However, under pathological conditions in which extracellular glutamate concentration rises to a high level (e.g. brain anoxia), presynaptic NMDARs may act to reduce glutamate release, thereby playing a protective role against neuronal death due to glutamate excitotoxicity.

Previous research reported that synaptic transmission and glutamate transporter activity at the calyx of Held synapse differ between physiological and room temperature [81]. Thus, the temperature-dependency of NMDAR-mediated presynaptic inhibition remains to be examined in order to further investigate the physiological and pathophysiological relevance. Besides, it needs to be noted that all data in this study were obtained using immature rats (P7–9). It is beyond the scope of our current study to clarify whether the NMDAR-dependent presynaptic inhibition is developmentally regulated, similar to TBOA-induced presynaptic mGluR activation [61].

In conclusion, the present study identified a novel regulatory mechanism for NMDAR-dependent presynaptic inhibition at an excitatory synapse in the auditory brainstem of rat pups by direct presynaptic recordings. Moreover, it also revealed the presence of functional presynaptic NMDARs. These findings bring significant insights to the controversial research field on presynaptic NMDARs. Finally, this study not only provides applicable implications to presynaptic inhibition at other central synapses, but also potentially serves to develop drugs targeting presynaptic NMDARs in the CNS.

## Supplementary Material

Supporting data

## Data accessibility

The datasets supporting this article have been uploaded as part of the electronic supplementary material.
